# The Gut Microbiome and Type 2 Diabetes Mellitus: Discussing A Complex Relationship

**DOI:** 10.3390/biomedicines8010008

**Published:** 2020-01-07

**Authors:** Angelos K. Sikalidis, Adeline Maykish

**Affiliations:** Department of Food Science and Nutrition, California Polytechnic State University, San Luis Obispo, CA 93407, USA; amaykish@calpoly.edu

**Keywords:** gut health, inflammation, insulin resistance, microbiome, microbiota, type 2 diabetes mellitus

## Abstract

Type 2 diabetes mellitus (T2DM) is a disease that affects over 9% of the United States population and is closely linked to obesity. While obesity was once thought to stem from a sedentary lifestyle and diets high in fat, recent evidence supports the idea that there is more complexity pertinent to the issue. The human gut microbiome has recently been the focus in terms of influencing disease onset. Evidence has shown that the microbiome may be more closely related to T2DM than what was originally thought. High fat diets typically result in poor microbiome heath, which then shifts the gut into a state of dysbiosis. Dysbiosis can then lead to metabolic deregulation, including increased insulin resistance and inflammation, two key factors in the development of T2DM. The purpose of this review is to discuss how microbiome relates to T2DM onset, especially considering obesity, insulin resistance, and inflammation.

## 1. Introduction

An estimated 39.8% of the US population is considered obese, as defined by the body mass index (BMI), hence a BMI > 30.0 [1]. This includes 35% of people under 40 and over 40% of middle-aged adults, and these numbers are only on the rise [2]. Obesity is a global epidemic interestingly seen both in developed and developing countries [2]. Unhealthy, often hypercaloric diets and sedentary lifestyles, typically coinciding from a behavioral standpoint, have been deemed major contributors to the development of the obesity epidemic in America and the world. Obesity can, over time, lead to various health complications, such as heart disease, high blood pressure, and type 2 diabetes mellitus (T2DM). While obesity has historically been thought of as the result of external factors, there is evidence to suggest that a significant component of obesity may be within a person’s own gut. Recent research on the human microbiome supports the notion that an individual’s microbiome profile could favor obesity, inflammation, and insulin resistance, eventually inducing T2DM.

The human microbiome is comprised of two primary phyla, namely Bacteroidetes and Firmicutes, typically in a ratio favoring Bacteroidetes over Firmicutes (B/F > 1). However, several studies have shown that in obese individuals, this ratio is altered, resulting in a higher prevalence of Firmicutes to that of Bacteroidetes [3,4]. Research has also demonstrated that transplanting microbiota from obese mice to germ-free (GF) mice resulted in significant weight gain in the latter compared to controls [5], suggesting the B/F ratio difference could contribute significantly to obese phenotype development. It is proposed that the specific demography of the gut microbiome in obese individuals causes increased energy harvest by the host organism, with any surplus leading to an overall significant increase of adiposity [6]. This function of the gut bacteria is likely attributed to the increased presence of Firmicutes, which have the ability to metabolize insoluble carbohydrates resulting in a higher energy harvest. The specifics of such a link and the exact mechanism remain largely elusive [5,7]. Nonetheless, the evidence suggesting a favorable link between the microbiome as per its demography and obesity is substantial, making the investigation of the microbiome and by extension its role in T2DM an interesting field of inquiry with potential therapeutic applications. The purpose of this review is to discuss the evidence pertaining to the relationship between the microbiome and T2DM, with concurrent consideration of the obesity, inflammation, and insulin resistance axes.

## 2. The Microbiome

In order to gather data regarding the microbiome, stool samples are typically collected and then analyzed using 16S rRNA sequencing. Via this approach, it has been estimated that human gut microflora contains over 35,000 species, and over 10 million non-redundant genes [7]. The species identified, fall into one of six major phyla—more specifically: Firmicutes, Bacteroidetes, Actinobacteria, Proteobacteria, Fusobacteria, and Verrumicrobia. Of these six, the two most common are Firmicutes and Bacteroidetes, consisting of 90% of the microbiome. Both Bacteroidetes and Firmicutes have been linked to obesity and thus will be the primary focus of the current review.

The Bacteroidetes phylum consists of four major classes, namely, Bacteroidia, Flavobacteria, Sphingobacteria, and Cytophagia, all of which have a role in fermenting otherwise indigestible carbohydrates [8]. Within these four classes, the most commonly found genera are *Sphingobacterium, Bacteroides, Tannerella, Parabacteroides, Alistipes*, and *Prevotella*, all of which are Gram-negative [9]. In humans, dietary fibers and fructooligosaccharides are indigestible in the sense that humans do not produce the enzymes for the digestion of these compounds [10]. However, bacteria present in the gut can ferment these compounds for their own benefit, releasing short chain fatty acids (SCFAs) within the host (gut environment) in the process [11]. This process can be beneficial for humans from an energetics perspective, as SCFAs can constitute a significant source of energy [7]. Firmicutes also play an important role in the generation of SCFAs, the main SCFA produced being butyrate (some Bacteroidetes also produce butyrate) [12,13]. The Firmicutes phyla can be grouped into three major classes: Clostridia, Negativicutes, and Bacilli. Firmicutes consist of over 200 genera, including *Staphylococcus, Lactobacillus, Ruminococcus*, and *Clostridium* [9]. The Firmicutes phyla consists primarily of Gram-positive bacteria, with the exception being those in the class Negativicutes. Negativicutes are interesting because of the presence of an outer membrane with lipopolysaccharides, making them stain Gram-negative [14]. The Firmicutes phyla has not been thoroughly investigated, and therefore a comprehensive understanding regarding its benefit to the human body remains elusive and largely non-conclusive as of yet. Nevertheless, the Firmicutes population is clearly positively associated with dysbiosis, with lower numbers of Firmicutes considered more ideal. The focus of most microbiome studies centers around decreased numbers of Firmicutes, derived from an observational standpoint, thus leaving a knowledge gap regarding Firmicutes’ function and mechanism(s) of action. However, it is believed that their primary role lies in metabolic degradation of an energy source [15] and can therefore be associated with calorie bioavailability and utilization. Firmicutes have also been found to raise lipid droplet numbers in zebrafish, which positively correlates with fatty acid uptake [16]. This is important, as fatty acids can store energy in the body to be used when glucose is not available [17] and have proven health benefits in certain cases depending on the type, such as a decreased risk of heart disease, cancer, and arthritis [18,19]. The issue with Firmicutes and metabolic deregulation has arguably to do with the degree of accumulation leading to significant lipid amount stored. In a healthy individual, the low number of Firmicutes present results in adequate energy uptake of the host that does not lead to high calorie availability, thus disfavoring positive energy balance. Overabundance of Firmicutes, in contrast, results in increased energy harvest, a higher caloric bio-availability, positive energy balance, all eventually promoting weight gain [7]. While this is not known for certain, it has been proposed as a biologically plausible and reasonably probable mechanism via which Firmicutes play a role in weight gain and eventually the development of obesity over time.

### Eubiosis vs. Dysbiosis

**Figure 1:**
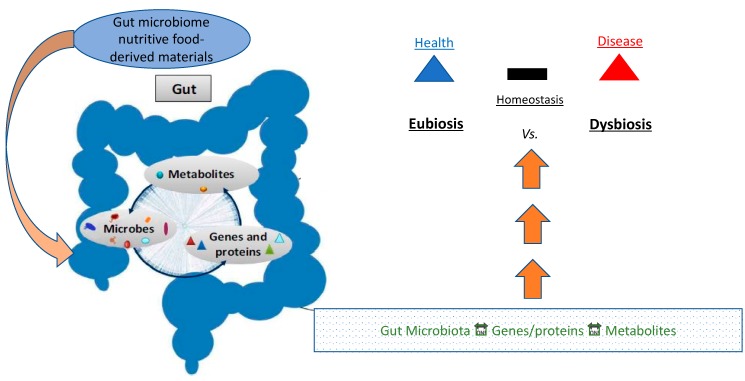
Conceptual schematic pictogram illustrating the relationship axis of microbes, metabolites, and gene expression vs. eubiosis/dysbiosis balance and disease risk.

Eubiosis (from Greek ευ/eu: good and βίος/bios: life) refers to the normal/healthy profile of gut microflora, as opposed to dysbiosis (from Greek δυς/dys: non-favorable/difficult and βίος/bios: life) which produces a demography that induces risk for certain diseases (Figure 1).

**Figure 2:**
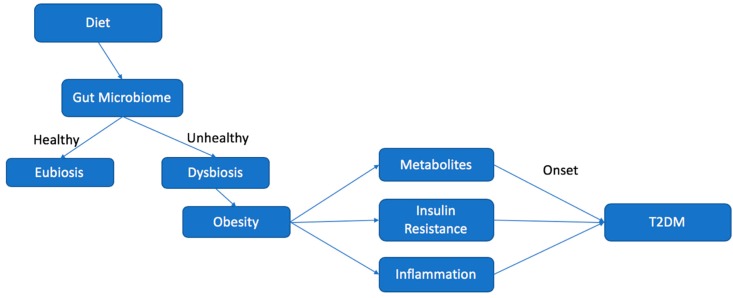
Chart illustrating the effect of diet on the microbiome and the relationship leading to increased risk for the development of type 2 diabetes mellitus (T2DM).

In a state of eubiosis, the microbiome plays several roles in producing SCFAs, branched chain amino acids (BCAAs), affecting lipid metabolism, and generating other key metabolites. Eubiosis in the gut typically is a condition in which there is a vibrant gut bacterial population composed of 95% Bacteroidetes, and 5% Firmicutes, forming an ideal B/F ratio. When the B/F ratio is such, the microbiome extends optimized gut health, regulates and controls opportunistic pathogens, and contributes to the entire body’s good health. [20]. Dysbiosis, on the other hand, can be defined as any change in the normal/desirable flora in an otherwise healthy gut [20]. In the vast majority of cases, dysbiosis has a negative impact, and can lead to obesity and the onset of several diseases, typically of chronic nature, including T2DM and ensuing CVD (Figure 2) [20].

In a groundbreaking experiment by Turnbaugh et al., germ-free (GF) mice were colonized with gut microbiota from conventionally raised (CONV) mice and were monitored for changes in weight. Within 10–14 days, the GF mice displayed increased body fat, despite a decrease in food consumption [5]. This change was shown to be attributed to the microbial fermentation of undigestible polysaccharides, absorption of monosaccharides, and genes in the microbiome that promote the growth of adipocytes. These findings led researchers to support that obese individuals are typically more efficient at energy harvest, by means of their microbiome, compared to lean counterparts, which may provide some explanation for weight gain.

In another set of experiments by Liou et al., diet-induced obese mice underwent Roux-en-Y gastric bypass (RYGB), which resulted in a decreased body weight and loss of fat mass. Standard 16S ribosomal sequencing was performed on fecal samples to study changes in the mouse microbiome after surgery. Mice that underwent RYGB displayed altered gut microbiota, especially by *Clostridiales*, a family in the Firmicutes phyla [21]. By the end of 12 weeks, the number of *Clostridiales* decreased significantly compared to pre-surgical numbers. This correlates with the variable B/F ratio, where lean individuals displayed lower numbers of Firmicutes than their obese counterparts [21]. This study shows that it is not only the microbiome itself that is responsible for weight fluctuation, but also the ideal B/F bacterial composition of the microbiome.

In a separate study Lund et al. fed CONV-raised mice and GF mice either a high fat diet (HFD) or low-fat diet (LFD). All mice were then monitored over the course of 2, 6, or 16 weeks for changes in weight. Weight monitoring interestingly revealed that the CONV mice fed the HFD displayed significant weight gain, as opposed to the GF mice fed the HFD, where the mice gained little to no weight. This points towards the idea that microbiome balance disturbance contributes to weight gain [22]. Taken together, these findings importantly suggest that dysbiosis within the microbiome of an HFD feeding regime is a contributor towards weight gain and not a consequence of it.

## 3. Major Metabolic Contributors to Microbiome Profile Identity (SCFA, BCAA, LPS)

It is well established that gut microbiota is responsible for the fermentation of otherwise indigestible carbohydrates, a byproduct of this process being SCFAs. Typically, SCFAs play an important role in protecting the gut, where they line the epithelium and help form tight junctions between cells preventing intestinal permeability. When the B/F ratio is altered, the proteins that form the junctions are reduced, resulting in potential lipopolysaccharide (LPS) translocation. Translocation of LPS is an important first step in triggering the immune response. LPS can then bind to toll-like receptor 4 (TLR4), resulting in activation and dimerization of the two [23]. Once this dimerization occurs, downstream adaptor molecules are recruited, activating IL-1 receptor associated kinase, tumor necrosis factor (TNF) receptor-associated factor, transforming growth factor B-associated kinase, c-Jun N-terminal kinases (JNK), and IκB kinase (IKK). This activation of JNK and IKK can also induce insulin receptor substrates (IRSs) serine phosphorylation, an important step in establishing insulin resistance [23]. The newly formed IKK complex then meets nuclear factor kappa-light-chain-enhancer of activated B cells (NF-κB) and activates it. The complex is then tagged for degradation. Then, NF-κB is in turn translocated into the nucleus, activating the inflammatory response [23].

### 3.1. LPS

LPS translocation is considered one of the first steps in the pro-inflammatory cascade response. To further emphasize the importance of dysbiosis, LPS, and inflammation, Whelan et al., fed mice either a high fat diet, a diet supplemented with LPS (a low dose), or a control diet. The mice fed LPS developed obesity in a similar way as those that were fed a high fat diet. However, when mice missing CD14, an immunoprotein responsible for inflammatory reactions, were fed LPS, no weight gain was observed [24]. In both the HFD and the diet supplemented with LPS, the binding to TLR-4 was able to occur. However, in the absence of CD14, the inflammatory response was never initiated. Similar results have been obtained in mice not expressing TLR-4 [25].

### 3.2. Short Chain Fatty Acids (SCFAs)

As previously mentioned, SCFAs are highly important in the regulation of the inflammatory response, and a decrease in Bacteroidetes results in a decrease of SCFAs. Butyrate, a type of SCFA, is a major metabolite and important for gut health. While its role is not completely understood, its known importance is highlighted by a series of experiments. In a study by Gao et al., mice were given sodium butyrate as a dietary supplement. Their insulin sensitivity and energy metabolism were both monitored over the course of 16 weeks. It was observed that mice which consumed an HFD but were given butyrate supplements did not develop insulin resistance or obesity [26].

Interestingly, a study explored the gut microbiota of urban Italians versus a community of hunter-gatherers called Hadza, located in Tanzania, analyzing how the former’s gut microbiota compares to that of a foraging lifestyle, one that all human ancestors took part in. Fecal samples from 27 Hadza and 16 Italians were analyzed, and while much of this study focused on microbiota demographics, SCFA profiles were also analyzed. Conclusively, it was found that urban Italians generate significantly more butyrate, whereas Hadza generate more propionate [27]. This is particularly interesting, as butyrate is typically associated with Firmicutes, and propionate with Bacteroidetes [27], but excess Firmicutes are associated with weight gain. Based on this association, it could be argued that butyrate supplementation in the discussed study would not be beneficial. However, by the same token it can be argued that this further emphasizes the importance of SCFAs. Even in adverse conditions, the phenotype is still improved, showing that SCFA functions in a corrective way in the gut. It is also important to note that some Bacteroidetes produce butyrate as well, meaning that in an ideal B/F ratio, the butyrate producing Bacteroidetes do produce ample butyrate to compensate for the lack of Firmicutes, thus restoring, at least partially, a metabolite balance in the gut environment [12].

Based on current knowledge, the microbiome seemingly plays an important role in inflammatory responses, both in its own right as well as in an interplay with the diet [28]. Mackay and colleagues studied colitis in GF- and CONV-raised mice. The mice were treated with DSS, to chemically induce colitis. The GF mice faired significantly worse than the CONV mice, displaying much worse colonic inflammation. Additionally, when GF mice were then colonized with CONV gut microbiota, their inflammation was reduced. To identify the cause of this reduction in inflammation, GF mice that were not colonized were treated with acetate, a SCFA, and known to be produced by Bacteroidetes. This also caused a decrease in colitis symptoms, further emphasizing the importance of SCFA in the inflammatory response [29]. This again underscores the importance of SCFA, especially those produced by Bacteroidetes, and argues in favor of the proposition that SCFA production is a plausible mechanism for salvaging desirable phenotypes as per the gut health and related metabolism. Furthermore, numerous studies have indicated that the gut environment is highly responsive to a variety of bioactive compounds found in food items (typically fruits and vegetables) in ways that reduce risk for several chronic diseases, including T2DM, CVD, and cancers [30].

### 3.3. Branched Chain Amino Acids (BCAAs)

While increased levels of SCFAs may be beneficial in the prevention of T2DM, this is not necessarily the case with BCAAs. Three of the nine essential amino acids are BCAAs (leucine, isoleucine, and valine) [31], and they must be obtained through diet in humans. Elevated levels of BCAAs have been observed in obese individuals and those with T2DM [32], while obese individuals demonstrate increased BCAA catabolism [33].

The effects of BCAA production and insulin resistance are fairly complex. BCAAs have been shown to interfere with insulin signaling by stimulating mTOR, a kinase complex that plays an important role in protein synthesis [34], S6K1, a kinase important for cell growth [35], and phosphorylation of insulin receptor substrate 1 (IRS1) [33,36].

To better understand this process, Newgard and co-workers fed rats an HFD, an HFD supplemented with BCAA, or an ND (normal diet; control). The rats were then fasted for 48 h, then re-fed their original diet. Upon re-feeding, there was an evident increase in the amount of phospho-mTOR^Ser2448^, phospho-S6K1^Thr389^, and phospho-IRS1^Ser302^ in the rats on the HFD supplemented with BCAA group, in comparison to the other two groups [33]. In the same study, the HFD/BCAA rats also demonstrated a lower food intake and less weight gain than the ND (control) rats but were equally as insulin resistant as those on the HFD [33]. This helps to illustrate the role of BCAA on the insulin signaling pathway, and how elevated levels of BCAA help to upregulate this pathway.

In a different study, involving human, 2422 normoglycemic individuals were followed for 12 years, 201 of which developed diabetes. The amino acids, amines, and other metabolites were observed initially and used as a baseline using liquid chromatography tandem mass spectrometry. Isoleucine, leucine, and valine exhibited higher concentrations during fasting, while levels were elevated up to 12 years prior to the development of T2DM. These observations correlated with a four-fold increase in the development of T2DM [37].

The results from the aforementioned studies are particularly interesting, as the recent shift in health trends interestingly contradicts what has been observed by experimentation. There has been an increasing emphasis on dietary supplements, probiotics, and overall heath outside of diet and exercise. A popular type of dietary supplement is BCAAs, where it is recommended to take them before or after weightlifting to build muscle mass and produce energy [38]. While the idea is on one hand to provide a series of essential amino acids, and on the other provide amino acids that induce protein synthesis, hence anabolism and in that sense support muscle growth, if this is not supported by exercise, significant results may not be seen. Furthermore, while anabolism is induced, so is the production of pro-anabolic hormones, such as insulin, or in any event create such orchestration of the metabolic signaling that maintains insulin input/signaling for longer. This, while it may induce anabolic processes, plausibly also induces insulin resistance over time [39,40]. If a person is also consuming enough protein in their diet, the supplementation of BCAAs is likely not necessary, and will not help the average person achieve better results, while it will arguably stress the kidneys needlessly making nephropathy more likely in a T2DM status [41]. Furthermore, looking at evidence produced by a series of studies discussed, it seems as if elevated levels of BCAAs may cause more anabolic induction leading to increased insulin resistance and the subsequent onset of T2DM.

## 4. Dysbiosis and the Development of T2DM

T2DM develops when, systematically, the pancreas is forced to produce gradually increasing amounts of insulin to achieve postprandial glucose clearance reaching a point of such low insulin responsiveness from peripheral tissues (insulin resistance) normoglycemia cannot be achieved [42]. The exact mechanism of this malfunction is unknown, however many factors, such as obesity, a sedentary lifestyle, genetics, diet, and other environmental factors, and now, the microbiome, seem to influence the onset and development of this disease [43]. Insulin postprandially stimulates cells to uptake glucose by binding to insulin receptor on cellar membrane initiating a signaling cascade that normally leads to the translocation of glucose transporter type 4 (GLUT4) to the cellular membrane, thus initiating glucose clearance, as GLUT4 transports glucose into the cell down a concentration gradient [44]. How precisely glucose undergoes this transportation is not entirely understood, while it is important in the attempt to control onset of T2DM. Once inside the cell, glucose is either used for energy production or stored as glycogen within specific cells (hepatocytes and myocytes). Notably, if insulin is not present, there is no effective alternative mechanism for glucose clearance, resulting in hyperglycemia [45].

All responses described above appear to be linked to the microbiome as well, while more specifically dysbiosis in the gut appears to be a risk factor for T2DM development. In a metagenome-wide study of 345 Chinese individuals with T2DM, 60,000 T2DM-associated markers were validated, and all correlated with gut dysbiosis, decrease in butyrate producing bacteria, and an increase in oxidative stress [46]. This pioneering study provided solid evidence to suggest that the microbiome plays an important role in the development of T2DM, and dysbiosis is a contributor to the disease.

### 4.1. Inflammation

There is no clear, direct, known pathway by which inflammation relates to T2DM, but there is increasing evidence supporting a definite relationship between induced inflammation and increased risk for insulin resistance, which in turn leads to T2DM. Individuals in a pre-diabetic state compensate for insulin resistance by β-cells insulin hypersecretion [47], but as the disease progresses, β-cells progressively grow less able to supply the needed amount of insulin, gradually become exhausted, and eventually die. In this context, β-cells dedifferentiation is being investigated as a means of β-cells failure in T2DM [48], but this pathway is not confirmed, while anti-inflammatory diets are considered to help reduce risk of diabetes [49]. Several inflammatory cytokines, such as IL-1, IL-6, NF-κB, and TNF-alpha, also have been linked to obesity. Specifically, IL-6 biosynthesis functions as an initial state of inflammation. Upon generation, it moves to the liver triggering the rapid protein synthesis of C-reactive protein (CRP), which will be discussed further later. IL-1 inhibits β-cell function by inducing the destruction of β-cells hence reducing β-cell mass over-time, which is primarily seen in T2DM development at the late stages of the disease. Higher levels of IL-1 have also been commonly observed in obese individuals. TNF-alpha IL-6, IL-1, are all adipokines, a subset of cytokines. They are secreted by adipose tissue and can function as pro-inflammatory signaling agents. As a result, dysregulation has been linked to obesity and T2DM onset, especially considering inflammation. In obese individuals, it has been consistently observed that expression of pro-inflammatory cytokines is subsequently commonly followed by insulin resistance as well [50], hence making cytokines an important area of investigation when considering T2DM risk, onset, and disease management. Based on this approach, the microbiome and inflammation have been a focus of study when looking for causes and treatments regarding obesity and T2DM.

The effect of RYGB on mice microbiomes was previously illustrated as an example [21], but such an effect on human microbiomes and the body as a whole is an important area of investigation for fully understanding how the microbiome and T2DM development are dynamically interrelated. In a study by Bornstein et al., five individuals with T2DM and one obese individual who had all undergone RYGB were studied for changes in microbiota as an effect of RYGB and how observed changes influenced disease management [51]. Researchers showed that the RYGB procedure resulted in a decrease in both Firmicutes and Bacteroidetes, and a concurrent increase in Proteobacteria. It is important to note that while Bacteroidetes decreased, the phylum was still present in higher amounts than in Firmicutes, and the ideal B/F ratio was actually more closely achieved towards desirable post-operation, suggesting a favorable effect of the surgical operation (RYGB) [51]. The RYGB and subsequent microbiota change in the body was also observed when association with the inflammatory state was tested. Out of all the detected species, 9 of the 22 species were significantly correlated to C-reactive protein, a biomarker for systemic inflammation commonly tested in the blood to assess inflammatory status [52]. Since nine bacterial species demonstrated a significant correlation with CRP levels in the blood, it was suggested that gut microbiome closely relates to the inflammatory state. Furthermore, a significant correlation of inflammatory state as assessed by CRP levels was seen with BMI, suggesting that a lower BMI correlates with a less inflamed state [48]. This is consistent with the proposition that obesity due to increased cytokine excretion induces a chronic mild pro-inflammatory state.

In a study investigating the effect of HFD on inflammatory markers, both CONV and GF mice were fed either HFD or ND for 16 weeks [22]. Intestinal inflammation was evaluated by observing TNF-α mRNA levels and activation of a NF-κB reporter gene. Both TNF-α and NF-κB are important in the activation and sustenance of inflammatory responses [22]. Results showed that CONV mice on the HFD demonstrated weight gain and upregulated TNF-α mRNA levels, but the same was not observed in GF mice. The TNF-α mRNA induction also directly preceded obesity onset in these animals. The same pattern was also observed for NF-κB activation, where it was activated in epithelia cells, immune cells, and endothelial cells in CONV mice. Furthermore, when fecal slurries from the HFD CONV mice were then used to inoculate GF mice, it was observed that this trans-inoculation was enough to activate NF-κB in GF mice [22]. These results again highlight the importance of the microbiome relative to the inflammatory response, suggesting that the inflammatory response activation can be mediated by the microbiome.

### 4.2. Insulin Resistance

While insulin resistance is a metabolic condition that typically eventually leads to T2DM, the microbiome appears to extend significant influence over the course of events and ultimately risk towards T2DM outcome. Work by Nieuwdorp et al., investigated the effects of microbial infusion in men with metabolic syndrome, defined as “a cluster of conditions that occur together… including increased blood pressure, high blood sugar, excess body fat around the waist, and abnormal cholesterol or triglyceride levels” [53]. Intestinal microbiota from lean donors were transferred to male recipients suffering from metabolic syndrome, and recipient microbiota and glucose metabolism were monitored post-transfer. Six weeks after trans-inoculation, insulin sensitivity of the recipients almost doubled, suggesting significant improvement in metabolic syndrome, while desirable butyrate producing microbiota also increased significantly [54].

Moreover, in a recent study, 291 non-diabetic Danish individuals underwent microbiome analysis, with their results being compared to those of 75 individuals with T2DM [55]. After analysis, insulin resistance levels and metabolic syndrome metabolites were investigated and compared between the two groups of focus [56]. The microbiome composition of both groups was then clustered based on metabolite production, where it was found that 19 of the 74 clusters were significantly associated with insulin resistance and metabolic syndrome. The correlated clusters were consistent across all 291 individuals and were also confirmed in the T2DM patients [55]. This suggests that certain metabolites produced by microbial clusters are strongly associated with higher insulin resistance, reinforcing the idea that certain microbiome configurations contribute to the development of insulin resistance.

Metformin is commonly prescribed medication to help manage T2DM, where it functions to suppress glucose production and increase insulin sensitivity. In a study evaluating the effects of metformin on metabolic improvement and the microbiome, mice on HFDs were evaluated. Mice were fed: (i) an HFD, (ii) an HFD and then switched to an ND, or (iii) an HFD supplemented with metformin. These dietary regimes were provided to induce obesity; hence obesity was the goal point, and not the development of T2DM. Results showed that upon administration of metformin to the HFD mice, the number of Bacteroidetes increased significantly, from 43% in the HFD group to 77% in the HFD–met group. Additionally, 18 metabolic pathways were also upregulated as a result of metformin administration [56]. While metformin is used primarily because of its positive effect on insulin sensitivity, interestingly it is shown to also alter the microbiome significantly in a desirable fashion. It cannot be ruled out that one of the potential mechanisms via which insulin sensitivity is improved upon metformin administration is mediated by metformin-induced changes in the microbiome.

While the direct connection between the microbiome and insulin resistance is not clear, it is evident that the microbiome plays an important role in regulating insulin resistance. These discussed findings provide a foundation for understanding this pathway, although more work needs to be done in the field to elucidate potential mechanistic pathways and series of events establishing how metformin may be influencing the microbiome leading to improved insulin sensitivity.

### 4.3. Oxidative Stress

The human body naturally produces free radicals when exposed to outside agents, such as food, alcohol, and air pollutants [57]. Reactive oxygen species (ROS) form as a result of metabolism, and transfer unpaired electrons causing oxidation of cellular machinery [47]. In a healthy individual, antioxidants, to a large extent, counter this process, neutralizing ROS and hence defending body homeostasis [58]. Imbalance, due to ineffective antioxidant defense, results in oxidative stress, which is closely related to glycation phenomena and diabetes onset [59]. A sedentary lifestyle and Western-type diets have been associated with overabundance of glucose and fatty acids, resulting in excess ROS. Glucose also reacts with plasma proteins to form glycation end-products, again producing ROS [59]. Oxidative stress induces inflammation, which in turn increases the risk for T2DM among other pathologies.

A recent study aimed to further understand the association between the microbiome and oxidative stress examining mice on HFDs [60]. Mice were either fed an HFD or HFD supplemented with lipoic acid, an antioxidant known to decrease oxidative stress [61]. ROS and total antioxidant capacity were assessed, as well as the microbiome of all mice in the study. Interestingly, in the mice supplemented with lipoic acid, lactobacilli were present in much lower numbers than in the mice on the HFD with no lipoic acid supplementation group. This constituted an important finding, as lactobacilli are members of the Firmicutes phylum. Thus, low numbers of lactobacilli observed also corresponded with decreased oxidative stress and better ROS levels, suggesting that antioxidants can ameliorate the microbiome profile and subsequently oxidative stress, and hence lower the risk for associated chronic disease such as T2DM [61].

While the correlation between the microbiome and onset of T2DM appears strong, there are several aspects of the microbiome that influence the development of disease. More specifically, microbiomes of patients with T2DM have begun to be evaluated in an attempt for a new search treatment. It has been revealed that the microbial composition of T2DM patients is quite different compared to non-T2DM individuals. The importance of diet in combination with disease state is critical in the establishment of a microbiome’s demography. As such, lifestyle and dietary intake factors need to be considered when evaluating the microbiome, in addition to disease state and medication. In a 2010 study, a group of 36 men, half of which had T2DM, with a wide range of BMI, underwent gut microbiota analysis [60]. Bacterial composition was analyzed using 16S rRNA sequencing, and it was found that the diabetic patients had significantly less Firmicutes present than their non-diabetic counterparts, specifically of the class Clostridia. Additionally, T2DM patients also displayed a higher B/F ratio (higher Bacteroidetes to Firmicutes) [60]. These results taken together may appear surprising at first, as one would believe the opposite to be true after considering the literature as a whole. However, lifestyle and diet were not considered in this study. Commonly, T2DM patients must follow a strict diet, low in simple carbohydrates and refined sugars, rich in complex carbohydrates, and low glycemic index foods/meals [62], whereas non-diabetic individuals are typically not on as strict of a dietary regime. The improved B/F ratio and overall lower numbers of Firmicutes observed, may be attributed to differences in the dietary regime plausibly followed by T2DM patients, as well as medication effects.

Overall, the thus far available evidence underlines a clear relationship between the type and state of the microbiome and the onset of chronic diseases, including T2DM. Further investigation considering the microbiome as a target for treatment towards chronic disease, particularly T2DM and ensuing CVD, is important and potentially highly valuable. The food industry and healthcare industry need to be involved in the development of potential foods [62] or systems [63] to provide potential therapeutic solutions enhancing and/or positively modifying the microbiome’s profile into an optimal, desirable state that would minimize risk of disease.

## 5. Concluding Remarks

It becomes obvious that the microbiome is evidently linked to obesity and subsequently T2DM onset. Therefore, it emerges as a significant target for treatment and prevention of disease and should hence constitute an area of focus for both the food and healthcare industry preferably in combined efforts through the development of novel therapeutic foods optimizing gut demography and thus maximizing the capitalization on the microbiome’s potential to extend health benefits pertinent to obesity and T2DM.

The so termed “fad” diets are certainly not of new news. Keto-diets, juice cleanses, and the Atkins diet are just a mere of three examples out of the numerous diets claiming to support a person to lose weight fast (interestingly this being the main argument and/or “selling point” for any “fad” diet as opposed to health promotion in its own right, for instance). Recently, the “Microbiome Diet (MD)” has come into play, a term coined by Dr. Raphael Kellman, and has been increasingly gaining visibility and has become somewhat trendy. The MD as a dietary approach is claimed to restore gut health (promoting eubiosis), increase metabolism, and decrease inflammation. It runs in three parts, with the overarching idea of eating less processed foods and more foods rich in prebiotics. Dr. Kellman has published a book describing the diet in its entirety, with testimonies by individuals who have followed it, certainly granted that these constitute simply anecdotal evidence. It is, however, important to note that it is challenging to identify published research on the effects of the diet described and published by Dr. Kellman himself (with the exception of his book) [64].

Probiotics have also been trending in recent years, again with emphasis on their effects on the microbiome. In a study done by the Cambridge Cardiac Care Centre in Ontario, Canada, the Dietary Approaches to Stop Hypertension (DASH) diet was investigated. Eighty individuals either consumed a diet typical of DASH, meaning a diet with emphasis on vegetables, fruits, and low-fat dairy items, or a DASH diet supplemented with probiotics. A total of 15% of these individuals were in the prediabetes stage. Hemoglobin A1C, fasting blood sugar levels, and blood pressure were all measured at the beginning and at the conclusion of a three-month dietary intervention experiment. Hemoglobin A1C tests, which provide a measure of plasma glucose concentration profile over time [65] and are used to evaluate the quality of glucose management, showed a decrease in A1C levels in both groups suggesting an improvement in glucose management, but the probiotic group was significantly lower compared to the non-supplemented group. In fact, the DASH-only group showed a decrease of 3.4%, but the probiotic-supplemented group showed a decrease of 8.9%, a significant difference. Fasting plasma glucose levels behaved similarly—10.7% decrease in the supplemented group, compared to a mere 3.3% in the DASH-only group [66].

While the Microbiome Diet and probiotic supplements are both somewhat new concepts, it does seem that there is significant scientific backing and mounting evidence to support their claims. While research regarding the Microbiome Diet itself is minimal, it is evident that decreasing that B/F ratio does make a notable difference concerning disease management. Probiotic supplements also extended a significant impact on health when paired with the DASH diet. As a result, it may be beneficial for both the food and healthcare industry to market towards the microbiome.

Recent research on the microbiome has caused a shift in focus on health and disease, highlighting the idea that eating a healthier diet reduces disease risk at levels as small as the microbiome. In fact, there have been several reports on other diseases triggered by obesity, such as heart disease and colon cancer. In a study done by Sikalidis et al., mice were fed a diet to induce obesity, and it was found that the obese mice had significantly more aberrant crypt foci and higher proliferation rate levels of colonocytes than their lean counterparts [67]. This further illustrates the importance of healthy diet and a healthy microbiome. Eating LFD improves the B/F ratio, decreasing risk of obesity, and therefore decreasing the risk of disease as a whole. The role of the microbiome in the onset of disease, in this case specifically T2DM, has been increasingly understood, resulting in investigation of this otherwise, until recently, relatively overlooked or underestimated factor with significant health implications. Onset of T2DM is much more complicated than addressing one particular factor, but by exploring the microbiome and how it modulates risk of T2DM, new answers and areas of research promoting more effective T2DM management could arise.

Diet is shown to extend a significant effect on gut health, and a healthy gut is responsible for more optimally regulating many pathways in the body. In the case of dysbiosis, many of these pathways are negatively impacted, contributing to the eventual onset of chronic disease. There is evidence supporting the idea that a plant-based diet results in decreased inflammation, a better B/F ratio, and an overall lower risk of disease [68]. Recently, however, the effect of cooked versus raw food on the microbiome has been investigated, with cooked or raw beef or sweet potato fed to mice. Both of the cooked diets resulted in increased body mass, despite a lower caloric intake, supporting the idea that cooking food results in an increased net energy gain [69]. Interestingly, the mice fed the cooked and raw beef diets both displayed similar microbial composition, but the mice fed the raw or cooked sweet potato displayed significant differences within their microbiome. The cooked sweet potato resulted in decreased diversity, and the mice fed the raw potato displayed increased weight loss. It is believed that this is due to the previously mentioned idea that cooking results in an increased energy harvest. The mice fed the raw potato diet also displayed better starch digestibility and degradation of plant compounds [69]. Therefore, it can be argued that while humans evolved to cook their food because of the beneficial energy gain, for weight loss and decreased risk of obesity a raw and plant-based diet may be more beneficial at least marginally. Interesting research is being done in the area of edible clay minerals as gut environment regulators for optimizing metabolism [70].

It is evident that there is a link between diet and the microbiome, including the microbiome’s role in obesity. There also appears to be a link between the microbiome and T2DM; however, further research on the subject is essential to determine a potentially effective treatment for T2DM, involving solutions utilizing the potential of the gut microbiome.
